# Integrating GPC3 with Other Biomarkers to Improve the Diagnosis of Early-Stage Liver Cancer

**DOI:** 10.3390/pathogens14121189

**Published:** 2025-11-21

**Authors:** Jing Xu, Lin Tan, Ning Jiang, Feng Zhang, Jinling Wang, Fengcheng Li, Jin Wang, Heng Li, Lichang Chen, Olivia Mezzetti, Wenyu Lin, Shasha Li, Yufeng Gao

**Affiliations:** 1 Department of Infectious Diseases, The First Affiliated Hospital of Anhui Medical University, Hefei 230022, China; fyey2563945@163.com; 2 Department of Hepatology, The Second People’s Hospital of Fuyang City, Fuyang 236015, China; 3 Department of Clinical Laboratory, The Second People’s Hospital of Fuyang City, Fuyang 236015, China; 4 Department of Hepatobiliary Surgery, The Second People’s Hospital of Fuyang City, Fuyang 236015, China; 5 Liver Center and Gastrointestinal Division, Department of Medicine, Massachusetts General Hospital, Harvard Medical School, Boston, MA 02114, USAwlin1@mgh.harvard.edu (W.L.)

**Keywords:** hepatitis B virus (HBV), glypican-3 (GPC3), hepatocellular carcinoma (HCC), GALAD, alpha-fetoprotein (AFP), Des-gamma-carboxy prothrombin (DCP)

## Abstract

Serum Glypican-3 (GPC3) levels in HCC patients are significantly higher than those in healthy individuals or patients with non-malignant liver diseases, making it a diagnostic marker for HCC. However, its diagnostic capability remains controversial due to its low sensitivity. The common marker AFP has limitations in terms of sensitivity and specificity, particularly in early-stage HCC. We sought to combine GPC3 detection with multi-biomarker panels to enhance sensitivity and specificity in early-stage HBV-, HCV-, and ALD-related liver cancer diagnosis. We applied receiver operating characteristic (ROC) analysis, which is used to evaluate the diagnostic performance of different biomarker tests, to develop comprehensive multi-biomarker panels that include GPC3, along with other biomarkers such as gender, age, AFP, AFP-L3%, and DCP, for assessment in the selected patients. We also applied univariate and multivariate logistic regression analysis to generate a specific diagnostic model for early HBV-induced HCC detection. We found that GPC3 levels in serum were significantly higher in HCC patients compared to CLD patients. We performed univariate and multivariate logistic regression analysis on the relevant indicators of early HCC to establish a new GDATA model for diagnosing early HCC. The new model included five indicators of early HCC: GPC3, DCP, AFP-L3%, TBIL and age. The diagnostic efficacy was better than that of GPC3, AFP, DCP and AFP-L3 alone. The diagnostic accuracy of the GDATA model for early HCC was significantly higher than that of the GALAD model or single indicators alone. The GDATA model thus provides a new promising diagnostic strategy for early HCC detection.

## 1. Introduction

Hepatitis B virus (HBV) infects nearly 300 million people globally [1,2,3,4] and chronic HBV (CHB) is a major cause of hepatocellular carcinoma (HCC) [5,6,7,8,9,10,11,12]. HCC is the fifth most common malignancy and the third leading cause of cancer death worldwide [13]. The mortality rate for HCC patients continues to rise [14]. More than 60% of HCC patients are already in the advanced stage when diagnosed and have lost the chance of early radical treatment, and the overall 5-year survival rate of HCC is only 20% [15,16,17]. Thus, early diagnosis is crucial to improve the prognosis of HCC patients. Alphafetoprotein (AFP) is the most widely used serological screening and diagnostic marker for HCC in clinical practice and is particularly accurate in diagnosing HCC when combined with other biomarkers, such as circulating tumor cell (CTC) count [18]. However, when used alone, its sensitivity and specificity in diagnosing early HCC and AFP-negative HCC cannot meet clinical needs [19]. Additionally, due to factors such as hepatocyte regeneration, inflammatory response, impaired liver function, and viral infection, some cirrhosis patients exhibit persistently elevated AFP levels, which significantly limits its clinical application [20,21,22]. Therefore, there is an urgent need to find new and non-invasive HCC serological diagnostic methods to improve the early diagnosis rate of HCC and the prognosis of HCC patients. The identification of new markers based on tumor-specific glycoproteins has become a research hotspot. Among them, glypican-3 (GPC3) has attracted much attention due to its unique expression pattern in HCC [23,24]. GPC3, as a cell surface glycophosphatidylinositol (GPI) anchored protein, belongs to the heparin sulfate (HS) proteoglycan family and plays an important role in regulating cell growth, differentiation and migration [25]. GPC3 is derived from the cell surface and expression is elevated in the tissues and serum of HCC patients but is low or absent in adjacent non-tumor lesions and benign liver diseases, suggesting its potential as a diagnostic marker for HCC [26,27,28]. Previous studies have shown that serum GPC3 has diagnostic value for HCC, but its specificity and sensitivity remain controversial [29,30]. A more detailed description of the clinical applications of different HCC and cirrhosis tumor markers can be found in Table S1. The current diagnostic model, the GALAD model, utilizes gender, age, AFP, AFP-L3% and DCP to screen for and diagnose HCC [31]. This model demonstrates high diagnostic efficacy across all stages of liver cancer. However, Huang et al. suggest that the model’s accuracy in diagnosing HCC may be affected by disease contexts and requires further evidence-based research to confirm [32]. In this study, we sought to evaluate the diagnostic accuracy of serum GPC3 levels for HCC (especially AFP-negative and early-stage HCC), and to generate a new model by combining GPC3 with other biomarkers to increase the accuracy of early-stage HCC diagnosis and thus improve the survival prognosis of patients.

## 2. Materials and Methods

### 2.1. Patients and Study Design

In this study, we adopted a prospective cohort design and recruited 100 HCC patients at the Fuyang Second People’s Hospital from March to November 2024 as the case group. All cases met the diagnostic criteria of the 2023 edition of the American Association for the Study of Liver Diseases (AASLD) “Practical Guidelines for the Prevention, Diagnosis, and Treatment of Hepatocellular Carcinoma.” HCC staging followed the Barcelona Clinic Liver Cancer (BCLC) staging system [33]. The inclusion criteria were as follows: aged 18 years or older, newly diagnosed with HCC, and not receiving any treatment for HCC. The average age of HCC patients was 60.85 ± 11.22 years. 75 patients (75%) were male, and 69% were early-stage HCC patients (BCLC stage 0/A). Patients with the following conditions were excluded: (1) taking oral anticoagulants; (2) combined with other organ malignancies; (3) had severe organ dysfunction. The control group included 100 patients with non-malignant chronic liver disease (CLD) who were treated during the same period. There were 50 patients with chronic hepatitis B and 50 patients with compensated hepatitis B-related cirrhosis. Their diagnosis met the criteria of the AASLD 2018 Guidelines for the Prevention, Diagnosis, and Treatment of Chronic Hepatitis B [34]. The average age of the control group was 48.80 ± 10.87 years, and 66 patients (66%) were male. The case and control groups in this study were comparable in key characteristics such as gender and etiology composition, so there was no need to use a matching design.

### 2.2. Sample Collection

General patient information, including age, gender, height (cm), and weight (kg) was recorded. 3–4 mL of fasting venous blood was collected from all patients in both the case group and control group, and was centrifuged at 1000× *g* for 15 min. The upper serum samples were collected and stored at −80 °C until detection.

### 2.3. Biochemical Examination and Analysis

Laboratory parameters obtained included white blood cells (WBC), platelets (PLT), platelet distribution width (PDW), albumin (ALB), globulin (GLB), albumin/globulin (A/G), alanine aminotransferase (ALT), aspartate aminotransferase (AST), γ-glutamyl transpeptidase (GGT), alkaline phosphatase (ALP), total bilirubin (TBIL), alphafetoprotein (AFP), electrolytes, hemoglobin, and hematocrit. Complete blood count (CBC) was measured using the SYSMEX CA5100 automatic clotting analyzer (Siemens Healthcare, Erlangen, Germany) [35,36,37,38]. Liver function tests (LFTs) were assessed using the Hitachi 7600 fully automatic biochemical analyzer (Hitachi High-Technologies Corporation, Hitachi-Omiya, Ibaraki Prefecture, Japan) [35,36,37,38]. Serum GPC3 was detected using the CUSABIO^®^ Human Glypican-3 (GPC-3) ELISA Kit (Catalog Number CSB-E11333h, CUSABIO Technology LLC, Wuhan, China), for which the detection principle and validity refer to the kit manual and prior validation studies [39]. Serum AFP, AFP-L3 and DCP were measured using the Beijing Rejing Biotechnology C2000 fully automatic chemiluminescence immunoassay and the corresponding detection kit (Beijing Hotgen Biotech Co., Ltd., Beijing, China). The standardized testing procedure follows the manufacturer’s protocol. All CLD patients underwent color Doppler ultrasound examination of the liver, gallbladder, and spleen (Diagnostic Ultrasound System and Transducers, Philips Ultrasound LLC, Bothell, WA, USA), and all HCC patients underwent upper abdominal MRI plain scan +g enhanced examination (MAGNETOM Skyra, Siemens Healthcare GmbH, Erlangen, Germany). The GALAD model score was calculated based on laboratory indicators. The calculation formula utilized is as reported below:

GALAD = −10.08 + 0.09 × age + 1.6 × sex (male is 1, female is 0) + 2.34 × Log10(AFP) + 0.04 × AFP-L3% + 1.33 × Log10(DCP), with AFP, AFP-L3, and DCP in ng/mL.

### 2.4. Statistical Analysis

SPSS22.0, GraphPad Prism 10, and MedCalc 20.027 statistical software were used for data analysis. NCSS-PASS software15 (NCSS LLC., Kaysville, UT, USA) was used to estimate sample size. There are no missing key indicators, and quantitative variables were directly included in the analysis using their original values. Data that met normal distribution were expressed as mean ± standard deviation, and comparison between two groups was performed using t tests. The measurement data that did not meet normal distribution were expressed as median and quartile M (IQR), and comparison between two groups was performed using Mann–Whitney U tests. The comparison between multiple groups was performed using Kruskal–Wallis H test. The count data were expressed as rate (%), and comparison between two groups was performed using the chi-square (χ^2^) test. The Delong test was used to compare the areas under the receiver operating characteristic curve (AUROC). Since there were no missing values in this study, and the core objective of this research was to explore the diagnostic value of GPC3 for HCC rather than to verify the robustness of the results in large-scale data, sensitivity analysis was not conducted. *p* < 0.05 was considered statistically significant. Univariate and multivariate logistic regression analyses were used to screen independent influencing factors associated with early HCC to generate an early HCC diagnostic model. The statistical methods described above refer to Wasserman’s All of Statistics [40].

## 3. Results

### 3.1. Patient Characteristics

**Figure 1:**
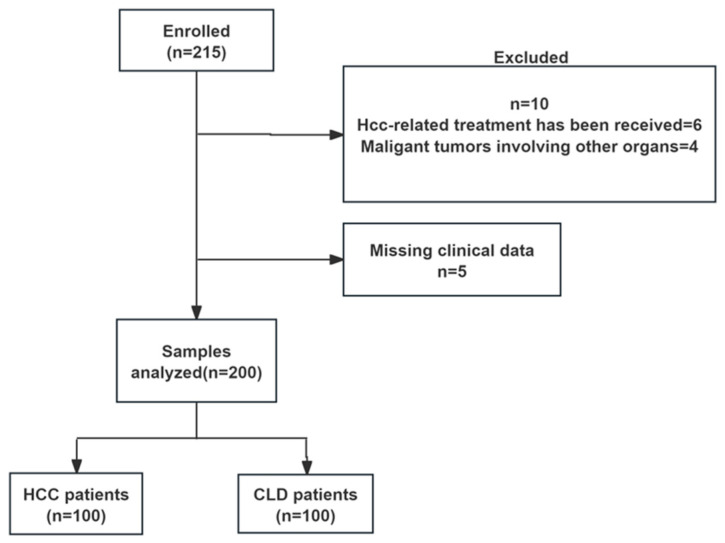
Study design and sample disposition flow chart. HCC, hepatocellular carcinoma; CLD, chronic liver disease.

Baseline patient demographics and clinical characteristics are presented in Table 1. A total of 200 patients were recruited in this study and assigned to the HCC group (100 cases) or the CLD group (100 cases). The research design and a sample distribution flow chart are shown in Figure 1. The average age of patients in the HCC group was higher than that in the CLD group (60.85 ± 11.22 years vs. 48.80 ± 10.87 years, t = 7.72, *p* < 0.001). Both groups were majority male, and there was no significant difference in gender distribution (*p* > 0.05). AST, TBIL, ALP, and γ-GGT levels in the HCC group were significantly higher than those in the CLD group, and ALB level was lower than that in the CLD group (all *p* < 0.05). HCC patient demographics and clinical characteristics are listed in Table 2. A total of 100 HCC patients were organized into the 0, A, B, C or D group according to BCLC staging. Early HCC patients (stage 0/stage A) accounted for 69%, stage B/stage C accounted for 19% and 12%, respectively. There were 62 patients (62%) with HCC with a diameter of ≤3 cm.

### 3.2. Analysis of Serum GPC3 Levels and Their Correlation with Clinical Characteristics of HCC

**Figure 2:**
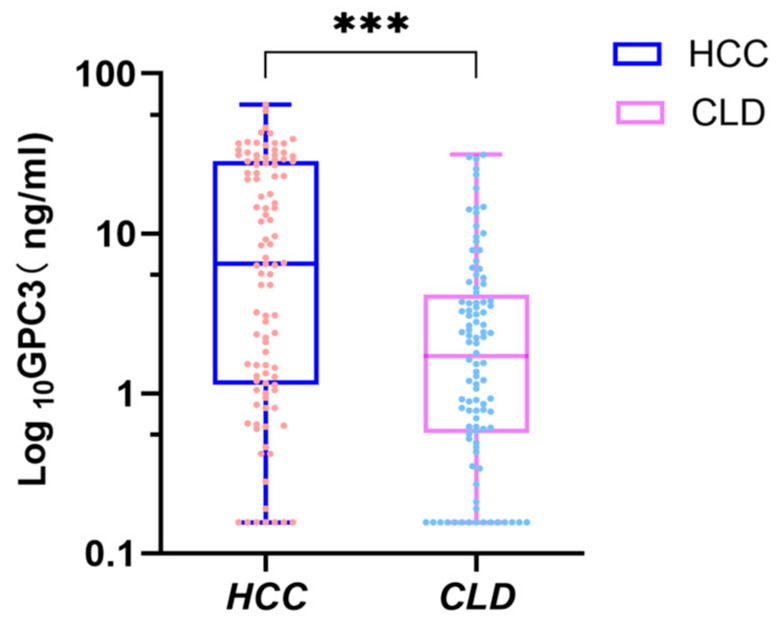
Serum GPC3 protein levels were significantly higher in hepatocellular carcinoma (HCC) patients compared to chronic liver disease (CLD) patients. GPC3 protein concentrations (ng/mL) in serum were measured by enzyme-linked immunosorbent assay (ELISA). (*** *p* < 0.0001).

We found that the serum GPC3 level of patients in the HCC group was 6.49 (1.14~28.28) ng/mL, significantly higher than that of the CLD group at 1.71 (0.57~4.16) ng/mL (Z = −4.630, *p* < 0.001) (Figure 2). HCC patients’ GPC3 levels were further analyzed based on age, gender, CHILD grade and clinical characteristics of the tumor. We found that serum GPC3 levels were not significantly associated with the patient’s age, gender, CHILD grade, tumor number, tumor size or the HCC metastasis status (Table 3).

### 3.3. Application of Serum GPC3 Value in the Diagnosis of Liver Cancer

**Figure 3:**
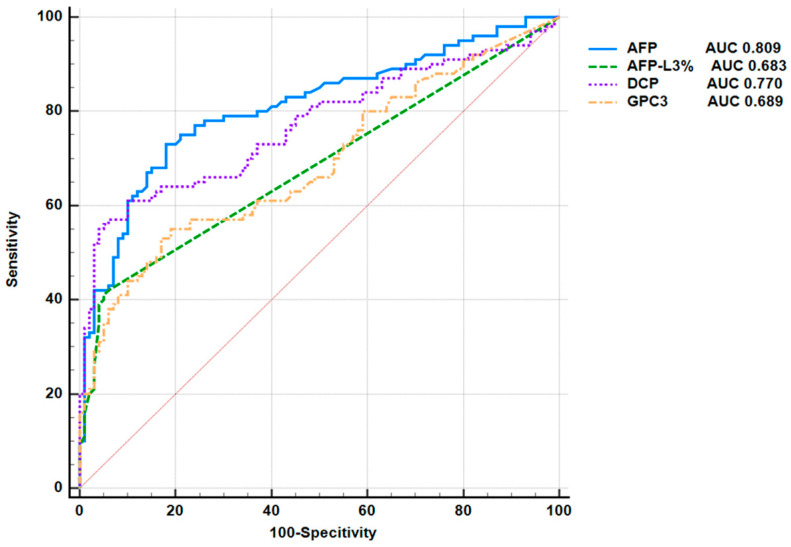
Comparison of ROC curves evaluating the diagnostic efficacy of AFP, AFP-L3%, DCP, and GPC3 for HCC. The ROC curve was used to represent the prediction model for HCC using the single clinical markers of AFP, AFP-L3%, DCP, and GPC3. Colors of curves correspond to biomarkers: blue (AFP), green (AFP-L3%), purple (DCP), and orange (GPC3).

The HCC patient group was set as the case group, and the CLD group was used as the control group. The receiver operating characteristic (ROC) curve was used to evaluate the diagnostic efficacy of serum GPC3 in HCC, and compared with that of AFP, AFP-L3%, and DCP (Figure 3). The area under the curve (AUC) of GPC3 in diagnosing HCC was 0.689 (95% CI: 0.620–0.753, *p* < 0.0001), and the AUC of AFP in diagnosing HCC was 0.809 (95% CI: 0.748–0.861), higher than that of GPC3 (*p* = 0.0151). The AUCs of DCP and AFP-L3% for diagnosing HCC were 0.770 (95% CI: 0.705–0.826) and 0.683 (95% CI: 0.614–0.747) respectively, which were not statistically significant compared with GPC3 (*p* = 0.0790 and 0.8982) (Figure 3). The optimal cut-off value, sensitivity, specificity and Youden’s index of GPC3 for diagnosing HCC are shown in Table 4. In the single biomarker index analysis, the optimal cut-off value of GPC3 was >5.53 ng/mL, with a sensitivity of 55%, a specificity of 81% and a Youden’s index of 0.36 for diagnosing HCC; the optimal cut-off value of AFP was >3.85 ng/mL, with a sensitivity of 73%, a specificity of 82% and a Youden’s index of 0.55; the optimal cut-off value of AFP-L3% was >6%, with a sensitivity of 42%, a specificity of 94% and a Youden’s index of 0.36 for diagnosing HCC; the optimal cut-off value of DCP was >18.98 ng/mL, with a sensitivity of 61%, a specificity of 90% and a Youden’s index of 0.51 for diagnosing HCC.

The analysis of combined biomarkers for diagnosis showed that the AUC of GPC3 and AFP for diagnosing HCC was 0.796 (95% CI: 0.734–0.850), with a sensitivity of 71%, a specificity of 81%, and a Youden index of 0.52. These biomarkers combined showed better diagnostic efficacy than the single marker GPC3 (*p* < 0.0001). When GPC3 was used in combination with DCP, the AUC was 0.803 (95% CI: 0.741–0.855), with a sensitivity of 67%, a specificity of 84%, and a Youden index of 0.51, which was also better than the diagnostic efficacy of GPC3 alone (*p* = 0.0001). When GPC3 and AFP-L3% were used in combination, the AUC was 0.792 (95% CI: 0.729–0.846), with a sensitivity of 68%, a specificity of 87%, and a Youden index of 0.55, which was also better than the AUC when GPC3 was used alone (*p* = 0.0002) (Table 4).

### 3.4. Efficacy of Serum GPC3 in Diagnosing AFP-Negative HCC

AFP ≤ 7ng/mL was used as the standard to determine AFP-negative HCC, a threshold that reflects the normal reference range for AFP in healthy individuals and effectively eliminates interference from physiologically mild AFP elevation in the context of chronic liver disease on diagnostic accuracy [41]. The efficacy of GPC3 in diagnosing AFP-negative HCC was evaluated and compared with the results of AFP-L3% and DCP detection, respectively. The AFP level in the control group was also ≤7 ng/mL. The AUC of GPC3 in diagnosing AFP-negative HCC was 0.681 (95% CI: 0.595–0.758, *p* = 0.0005), and the AUC of DCP in diagnosing AFP-negative HCC was 0.783 (95% CI: 0.704–0.849, *p* = 0.0005). Both are effective in diagnosing AFP-negative HCC, and there is no significant difference in diagnostic efficacy between the two (*p* = 0.1099). AFP-L3% is not evaluable due to being below the detection limit.

### 3.5. Efficacy of Serum GPC3 in Diagnosing Early HCC

**Figure 4:**
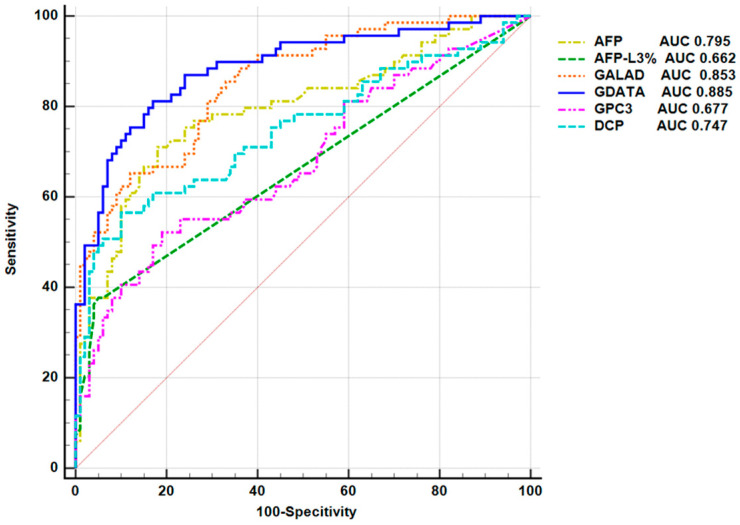
AUC curves for the diagnosis of early-stage HCC by the GDATA model, the GALAD model, GPC3, AFP, DCP and AFP-L3%. Colors of curves correspond to biomarkers: dark blue (GDATA), orange (GALAD), pink (GPC3), yellow (AFP), turquoise (DCP), and green (AFP-L3%).

In the HCC group, BCLC stage 0 and stage A were defined as early HCC, and stage B and stage C were defined as intermediate and advanced HCC, respectively. The efficacy of GPC3 in diagnosing early HCC was evaluated by ROC analysis. The results showed that the AUC of GPC3 for diagnosing early HCC was 0.677 (95% CI: 0.601–0.747, *p* < 0.0001), which was lower than the AUC of AFP (0.795) (95% CI: 0.726–0.853, *p* < 0.0001). The difference was statistically significant (*p* = 0.042), indicating that AFP was more effective than GPC3 in diagnosing early HCC. The AUC of AFP-L3% for diagnosing early HCC was 0.662 (95% CI: 0.586–0.733, *p* < 0.0001), which was not significantly different from GPC3 (*p* = 0.7928). The AUC of DCP for diagnosing early HCC was 0.747 (95% CI: 0.675–0.811, *p* < 0.0001), which was also not significantly different from GPC3 (*p* = 0.2015) (Figure 4). The AUC for combined GPC3 and AFP detection was 0.785 (95% CI: 0.716–0.845, *p* < 0.0001), significantly higher than GPC3 alone (*p* = 0.0002). Compared with the AUC of AFP alone for diagnosing early HCC, however, the difference was not statistically significant (95% CI: −0.0747–0.0931, *p* = 0.8298). The AUC for GPC3 combined with AFP-L3% was 0.795 (95% CI: 0.726–0.853, *p* < 0.0001), which was also superior to GPC3 alone (*p* = 0.0006) and comparable to AFP alone (95% CI: −0.0833–0.0836, *p* = 0.9973). The AUC for GPC3 combined with DCP was 0.779 (95% CI: 0.709–0.839, *p* < 0.0001), significantly higher than GPC3 alone (*p* = 0.0008), with no statistically significant difference compared to the AUC of AFP alone (95% CI: −0.0896–0.121, *p* = 0.7680). These findings suggest that GPC3 alone demonstrates moderate diagnostic efficacy for early HCC, while AFP alone exhibits optimal diagnostic performance. Notably, when GPC3 is combined with AFP, AFP-L3%, or DCP, the complementary advantages among these markers achieve diagnostic performance comparable to AFP alone, significantly enhancing the ability to distinguish early HCC.

### 3.6. Construction and Evaluation of an Early HCC Diagnostic Model

We performed the univariate and multivariate logistic regression analysis on the clinical data and serum tumor markers of early HCC patients to generate the new GDATA scoring model for diagnosing early HCC. Univariate analysis showed that age, WBC, ALB, AST/ALT, GGT, ALP, TBIL, AFP, LN (AFP-L3%), DCP, GPC3 and other variables may be associated with the risk of early HCC (*p* < 0.05). The positive variables in the above univariate analysis were further included in the multivariate logistic regression modeling. We found that after adjusting for confounding factors, GPC3, DCP, LN (AFP-L3%), TBIL and age were independent risk factors for the occurrence of early HCC (*p* < 0.05, Table 5). According to the results of multivariate logistic regression analysis, the early HCC diagnosis model was constructed with the following formula:

GDATA = −2.031 + 0.094 × GPC3 + 0.021 × DCP + 1.692 × LN(AFP-L3%) + 0.056 × TBIL + 0.08 × AGE

**Figure 5:**
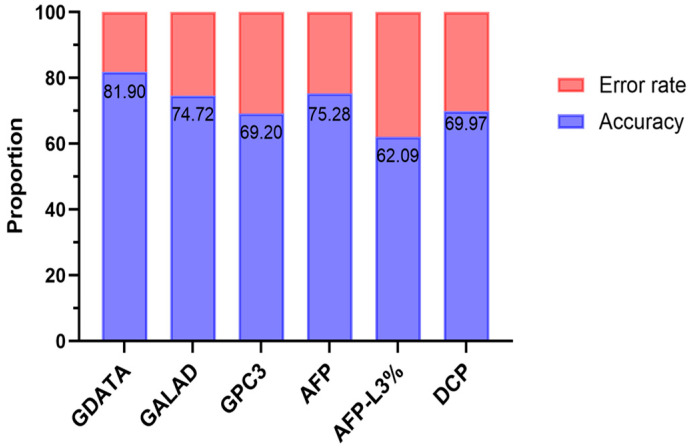
The accuracy of GDATA in diagnosing early-stage HCC. Accuracy is shown in blue and error rate in orange.

The GDATA diagnostic model was used to score the patients in the case group and the control group. ROC curve analysis based on the scores was performed to evaluate the efficacy of GDATA in diagnosing early HCC. The results showed that the AUC of GDATA was 0.885 (95% CI: 0.827–0.929, *p* < 0.0001), the Youden index was 0.6416, the sensitivity was 81.16%, and the specificity was 83.00%. The efficacy of GDATA in diagnosing early HCC was compared with the internationally recognized HCC screening and diagnosis model GALAD and serum markers GPC3, AFP, AFP-L3% and DCP (as shown in Figure 4). The AUC of the GALAD model for diagnosing early HCC was 0.853 (95% CI: 0.791–0.903, *p* < 0.0001). Compared with the GDATA model, there was no significant difference in AUC between the two models (Z = 1.232, *p* = 0.218), indicating that there was no significant difference in the efficacy of the GDATA model and the GALAD model in diagnosing early HCC. However, the AUC of the GDATA model for diagnosing early HCC was significantly higher than that of single tumor markers GPC3, AFP, AFP-L3% and DCP, indicating that the GDATA model has a higher discriminatory ability in the diagnosis of early HCC. Additionally, the GDATA model achieves a sensitivity of 72.46% at 90% specificity, higher than the GALAD model and single indicators alone. Even when specificity is increased to 95%, its sensitivity remains at 49.28%, surpassing all other indicators (Table S3). In terms of diagnostic accuracy, the GDATA model had an accuracy of 81.90% in diagnosing early HCC, which was significantly higher than the GALAD model (74.72%), as well as all single indicators (GPC3: 69.20%; AFP: 75.28%; AFP-L3%: 62.09%; DCP: 69.97%) (see Figure 5 for specific comparison results).

## 4. Discussion

HBV-induced HCC is a malignant tumor that poses a serious threat to human health, and early diagnosis is key to improving patient prognosis [42]. Currently, there is a lack of serum markers with both high sensitivity and high specificity for diagnosing early HCC. This study evaluated the efficacy of serum GPC3 in diagnosing HCC, AFP-negative HCC, and early HCC, and constructed a diagnostic scoring model for early HCC. It has previously been reported that GPC3 expression was not associated with age, tumor size, or vascular invasion, and that GPC3 expression in HCC tissue was also not correlated with patient age, gender, tumor size, tumor number, and distant metastasis [5,43,44]. This was supported by our findings, which showed that serum GPC3 level in the HCC group was significantly higher than that in the CLD group, and its level was not correlated with the age, liver function level, number of tumors, tumor size, or presence of metastasis in HCC patients, and there was no significant difference in the GPC3 level between male and female HCC patients (*p* = 0.6770). It has been suggested that serum GPC3 level in HCC patients is higher than that in cirrhosis patients [45]. Previous studies have also shown that the elevated serum GPC3 level in HCC patients is associated with the direct regulation of its transcription by c-Myc transcription factor [46]. Age-related physiological changes or sex-related differences in hormone levels may have little effect on the c-Myc-mediated transcriptional regulatory pathway, supported by the finding that serum GPC3 levels are not correlated with patient age or gender. The reasoning for the phenomenon that GPC3 levels are not correlated with tumor size, number, and distant metastasis has not been fully elucidated. It is speculated that the expression of GPC3 may be activated in the early stages of tumorigenesis and maintained at a relatively stable level without significant changes during tumor progression. However, the specific reasoning must be further explored in subsequent studies.

Regarding the efficacy of serum GPC3 and AFP in diagnosing HCC, Some reports suggested that the efficacy of AFP in diagnosing HCC (AUC = 0.83) is higher than that of GPC3 (AUC = 0.62) [47]. Other findings suggested that serum GPC3 and AFP have the same efficacy in diagnosing HCC [48]. An additional report suggested that the AUC of GPC3 for diagnosing HCC is only 0.519, which is not suitable as a serum marker for diagnosing HCC [49]. This study found that GPC3 does have diagnostic value for HCC, but its diagnostic efficacy is lower than that of AFP. The differences in the conclusions of previous studies may be related to the etiology of the study population, the clinical stage of HCC, and the differences in detection methodology. Previous studies have shown that the combined diagnostic strategy of different biomarkers can provide more accurate information for HCC diagnosis [50,51]. AFP, DCP, and AFP-L3% were significantly better than the AUC of GPC3 alone, indicating that the detection of a combination of markers can effectively improve the diagnostic accuracy of HCC. The sensitivity of GPC3 combined with AFP in diagnosing HCC was the highest among the three combined tests, suggesting that this combination may be more suitable for early screening of HCC to reduce the risk of missed diagnosis. When GPC3 is combined with AFP-L3%, the specificity is as high as 87%, which may be more conducive to excluding non-HCC patients and reducing the misdiagnosis rate. The AUC of GPC3 combined with DCP is 0.803, the highest among the three groups, and the specificity is 84%, indicating that its overall diagnostic efficacy is the highest. From the perspective of biological mechanisms, GPC3 and AFP, DCP, and AFP-L3% respectively reflect different pathological processes in different stages of HCC, such as cell proliferation, angiogenesis, and abnormal glycosylation. Combined detection can capture tumor heterogeneity from multiple dimensions, thereby improving diagnostic accuracy [52,53,54,55]. AFP-negative HCC accounts for more than 30% of all HCCs. Currently, there is a lack of effective and reliable diagnostic markers, and it is very easy to miss the diagnosis [2]. Our study found that GPC3 can be used as a diagnostic marker for AFP-negative HCC, and its diagnostic efficacy is comparable to that of DCP. Additionally, we found no significant correlation between the expression of GPC3 and the AFP status. GPC3 elevation can be detected in about 50–60% of AFP-negative HCC patients, which may be related to its specific activation in dedifferentiated tumor cells [39], while elevated DCP is caused by defective carboxylation of specific amino-terminal glutamic acid residues in HCC patients [56]. Although the two have different action pathways, they can both identify HCC independently of AFP. In this study, AFP-L3% did not show diagnostic value for AFP-negative HCC, which may be related to the low baseline AFP level in AFP-negative patients. AFP-L3% is the ratio of AFP isomers to total AFP. When total AFP is ≤7 ng/mL, even if the AFP-L3% ratio increases, its absolute value may still be lower than the detection threshold, resulting in limited clinical value. However, one previous study found that 50% of AFP-negative HCC patients tested positive for AFP-L3, and the AFP-L3 level was not related to the total AFP level. They found that the maximum AUC of AFP-L3 for diagnosing AFP-negative HCC was 0.6094, which differs from our results [57]. Perhaps due to the different stages of the patients enrolled and the different detection threshold settings, the AFP baseline levels of the samples in our study were lower, making it difficult to detect the absolute value of AFP-L3. Accurate diagnosis of early HCC is crucial for the selection of treatment methods for HCC patients and improving patient prognosis [8]. This study found that GPC3 has diagnostic value for early HCC, but its diagnostic efficacy is lower than that of AFP. It has been reported that in patients with BCLC stage 0 and stage A HCC, the positive rate of GPC3 (76.43%, 120/157) was significantly higher than that of AFP (64.33%, 101/157; *p* = 0.019), while no difference was detected in the positive rates between the two groups in patients with stage B and stage C HCC, indicating that serum GPC3 is superior to AFP as a sensitive marker for early HCC [58]. However, other findings suggested that serum GPC3 did not show diagnostic value for early HCC. The differences in these reports may be due to different staging criteria for HCC patients enrolled in the studies and different serum GPC3 detection methods [59]. The GALAD model is a model for diagnosing and screening HCC and was originally constructed based on five indicators: gender, age, AFP, AFP-L3% and DCP [31,60]. The follow-up study suggested that the AUC value of the GALAD model for diagnosing HCC was 0.97, which was significantly higher than the AUCs of AFP, AFP-L3% and DCP alone (0.88, 0.84 and 0.90, respectively, with *p* values < 0.05). The GALAD model also showed high diagnostic performance for early HCC (BCLC0/A stage), with an AUC value of 0.96, a sensitivity of 86% and a specificity of 89%. Some reports suggested that for the BCLC stage 0/A HCC population, the pooled sensitivity, pooled specificity, and estimated AUC of the GALAD score were 0.78, 0.80, and 0.86 (95% CI: 0.83–0.89), respectively [61]. Others report that, for early-stage HCC, the AUROC of single-time point GALAD was 0.78, and the AUROC of longitudinal GALAD was 0.83 [62]. At present, the advantages of the GALAD model in the diagnosis of HCC have been confirmed by clinical evidence and are recommended by many national guidelines for the screening and diagnosis of HCC. In this study, the AUC of the GALAD model for diagnosing early HCC was 0.853, the sensitivity was 65.22%, and the specificity was 88%, which also confirmed that the GALAD model has high value in the diagnosis of early HCC. Through logistic regression analysis, we established a new model for diagnosing early HCC—the GDATA model, which includes five indicators: GPC3, DCP, AFP-L3%, TBIL and age. Compared with the GALAD model, it has similar diagnostic efficacy in diagnosing early HCC. The GDATA model combines four specific serum biomarkers including GPC3 and total bilirubin that reflect liver metabolic conversion function. It has significant clinical advantages and integrates total bilirubin (TBIL, reflecting liver metabolic function), making up for the neglect of liver function status by traditional markers. In addition, the model does not include AFP, providing a new option for the diagnosis of HCC with low AFP expression. This innovation provides a new solution for the accurate diagnosis of early HCC and AFP-negative cases. The accuracy of the GDATA model in diagnosing early HCC is 81.90%, which is higher than the GALAD model (74.20%). In actual clinical applications, it can be combined with AFP to further improve the diagnosis rate of early HCC and the 5-year survival rate of HCC.

### Limitations

Limitations of this study include the fact that it is a single-center cohort study with a relatively small sample size. Additionally, the control group was mainly composed of patients with chronic liver disease, and there is a lack of data on healthy patients. The effectiveness of GPC3 in diagnosing early HCC in different contexts needs to be further verified. To address these limitations, future studies can include a wider range of HCC staging through multi-center, large-sample cohorts, and combine that with long-term follow-up data to further evaluate the clinical application value of the GDATA model. In conclusion, serum GPC3 has diagnostic value for HCC, and combined detection of GPC3 with AFP, AFP-L3% and DCP can significantly improve the accuracy of HCC diagnosis. The GDATA model constructed by integrating GPC3, DCP, AFP-L3%, TBIL and age showed the same efficacy as the established GALAD model in the diagnosis of early HCC. Our findings thus provide a new strategy for early HCC diagnosis that could increase the early diagnosis rate and improve the prognosis of patients.

While the GALAD model demonstrates strong efficacy as a tool for HCC screening or diagnosis, it still has certain limitations. A recent phase 3 biomarker study prospectively compared the performance of GALAD scoring and alpha-fetoprotein (AFP) in early detection of hepatocellular carcinoma (HCC). The results showed that GALAD scoring exhibited significantly higher sensitivity than AFP at 12 months. However, since the study primarily involved patients with HCV or MASLD, its findings may not be generalizable to other etiologies, like HBV infection, or to Asian populations [63]. Huang et al. also noted that whether the diagnostic accuracy of the GALAD model is influenced by disease background requires further evidence-based research for validation [32].

## Figures and Tables

**Table 1:** General characteristics of patients in the HCC and CLD group.

Variables	HCC	CLD	Statistic	*p* Value
	(n = 100)	(n = 100)		
Age (years)	60.85 ± 11.22	48.80 ± 10.87	t = 7.72	<0.001 ***
Gender [n (%)]			X^2^ = 1.947	0.163
Male	75 (75)	66 (66)		
Female	25 (25)	34 (34)		
Etiology [n (%)]				
HBV	69 (69)	96 (96)	X^2^ = 25.247	<0.001 ***
HCV	2 (2)	0	-	0.497
ALD	2 (2)	0	-	0.497
HBV + ALD	21 (21)	4 (4)	X^2^ = 13.211	<0.001 ***
HCV + ALD	2 (2)	0	-	0.497
Others	4 (4)	0	-	0.497
Child-Pugh grade [n (%)]			X^2^ = 23.464	<0.001 ***
A (5–6)	79 (79)	100 (100)		
B (7–9)	21 (21)	0		
C (10–15)	0	0		
ALT (U/L)	29.00 (21.00~40.75)	23.00 (16.25~33.00)	Z = −2.705	0.07
AST (U/L)	32.50 (24.25~49.00)	22.00 (18.00~29.00)	Z = −5.901	<0.001 ***
TBIL (umol/L)	19.300 (13.700~26.450)	14.900 (11.200~20.775)	Z = −3.382	0.001 **
ALP (U/L)	101.50 (81.25~137.25)	79.00 (62.00~98.25)	Z = −4.900	<0.001 ***
GGT(U/L)	57.00 (30.00~99.50)	27.50 (19.00~48.00)	Z = −5.734	<0.001 ***
ALB (g/L)	42.300 (36.300~46.475)	46.000 (42.825~48.175)	Z = −4.750	<0.001 ***

Data are presented as a number and percentage, or mean ± standard deviation, or medians with interquartile range (IQR, 25–75%). ** *p* < 0.01, *** *p* < 0.001.

**Table 2:** Clinical Characteristics of HCC patients.

Characteristics	n (%)
Maximum tumor diameter (cm)	
≤2	33 (33)
>2, ≤3	36 (36)
>3, ≤5	17 (17)
>5, ≤10	10 (10)
>10	4 (4)
Number of tumors	
1	81 (81)
2–3	13 (13)
≥4	6 (6)
Vascular invasion/metastasis	13 (13)
BCLC stage	
0	29 (29)
A	40 (40)
B	19 (19)
C	12 (12)
D	0

**Table 3:** Correlation Analysis of GPC3 Levels and Clinical Characteristics of HCC.

Variables	GPC3 Level (ng/mL)	Statistical Value	*p*-Value
Age (years)		Z = −0.025	0.98
≤50	10.52 (0.77~28.21)		
>50	6.49 (1.15~28.45)		
Gender		Z = −0.752	0.452
Male	5.55 (1.00~28.29)		
Female	9.63 (3.15~26.38)		
Child–Pugh Classification		Z = −0.351	0.752
Class A (5–6)	7.04 (1.16~28.25)		
Class B (7–9)	6.44 (0.82~29.88)		
Tumor Size (cm)		Z = −0.243	0.808
≤3	7.04 (1.27~28.29)		
>3	6.30 (1.07~28.58)		
Number of Tumors		Z = −1.955	0.051
Single	8.51 (1.60~28.91)		
Multiple	1.47 (0.62~25.90)		
BCLC Staging		Z = −1.174	0.24
0/A	5.62 (1.11~23.86)		
B/C	11.91 (1.13~33.20)		
Metastasis		Z = −1.825	0.068
No Metastasis	6.30 (1.05~26.76)		
Vascular Invasion/Extra— Hepatic Metastasis	28.25 (2.66~32.03)		

**Table 4:** Diagnostic Performance of Four Serum Biomarkers for HCC.

Variables	AUC	Cut-Off Value	Sensitivity	Specificity	Youden Index
GPC3	0.689	>5.53	55%	81%	0.36
AFP	0.809	>3.85	73%	82%	0.55
AFP-L3%	0.683	>6%	42%	94%	0.36
DCP	0.77	>18.98	61%	90%	0.51
GPC3 + AFP	0.796	>0.39	71%	81%	0.52
GPC3 + DCP	0.803	>0.40	67%	84%	0.51
GPC3 + AFP-L3%	0.792	>0.46	68%	87%	0.55

**Table 5:** Univariate and multivariate logistic regression analysis for early-stage HCC.

Variable	Univariate Analysis		*p* Value	Multivariate Analysis		*p* Value
	OR	95% CI		OR	95% CI	
Age (years)	1.103	1.063–1.144	<0.001	1.084	1.031–1.139	0.002
Sex (male)	0.635	0.319–1.261	0.194			
WBC	0.786	0.649–0.952	0.014	0.795	0.563–1.122	0.795
RDW-CV	0.991	0.969–1.014	0.456			
PDW	0.954	0.806–1.130	0.586			
ALB	0.898	0.844–0.954	0.001	1.011	0.916–1.116	0.827
ALT	1.007	0.997–1.018	0.190			
AST	1.015	0.999–1.032	0.062	0.99	0.977–1.003	0.135
AST/ALT	2.253	1.188–4.270	0.013	1.88	0.848–4.168	0.12
GGT	1.009	1.002–1.017	0.011	1.007	0.997–1.016	0.175
ALP	1.012	1.003–1.021	0.004	0.994	0.980–1.008	0.403
TBIL	1.057	1.021–1.095	0.002	1.058	1.002–1.118	0.043
AFP	1.01	1.002–1.018	0.013	1.004	0.995–1.013	0.382
LN (AFP-L3%)	14.99	4.465–50.319	<0.001	5.432	1.160–25.427	0.032
DCP	1.034	1.010–1.085	0.005	1.021	1.003–1.040	0.022
GPC3	1.081	1.043–1.121	<0.001	1.098	1.044–1.155	<0.001

## Data Availability

The raw data supporting the conclusions of this article will be made available by the authors, without undue reservation.

## Supplementary Materials

The following supporting information can be downloaded at: https://www.mdpi.com/article/10.3390/pathogens14121189/s1, Table S1. Clinical application of different cirrhosis and hepatocellular carcinoma (HCC) tumor markers; Table S2. A comparison of the GDATA and GALAD models for HCC diagnosis; Table S3. Sensitivity of GDATA, GALAD, and single markers at 90% and 95% specificity.

## Abbreviations

The following abbreviations are used in this manuscript:

HBV	Hepatitis B Virus
GPC3	Glypican-3
HCC	Hepatocelllular carcinoma
AFP	Alpha-fetoprotein
DCP	Des-gamma-carboxy prothrombin
CHB	Chronic Hepatitis B virus
CTC	Circulating tumor cell
CLD	Chronic liver disease
GPI	Glycophosphatidylinositol
AASLD	American Association for the Study of Liver Diseases
BCLC	Barcelona Clinic Liver Cancer
WBC	White blood cells
PLT	Platelets
PDW	Platelet distribution width
ALB	Albumin
GLB	Globulin
A/G	Albumin/globulin
ALT	Alanine aminotransferase
AST	Aspartate aminotransferase
GGT	γ-glutamyl transpeptidase
ALP	Alkaline phosphatase
TBIL	Total bilirubin
CBC	Complete blood count
LFT	Liver function test
ROC	Receiver operating characteristic
AUROC	Area under the receiving operating characteristic curve
AUC	Area under the curve
HCV	Hepatitis C virus
ALD	Alcohol-related liver disease
GALAD	Gender, age, AFP-L3%, AFP, DCP
GDATA	GPC3, DCP, AFP-L3%, TBIL, age
