# Supplementary material for: Integrating GPC3 with Other Biomarkers to Improve the Diagnosis of Early-Stage Liver Cancer
Source: Pathogens. 2025 Nov 21;14(12):1189. doi: 10.3390/pathogens14121189 (PMC12736379; doi:10.3390/pathogens14121189)
Supplement: Supplementary file 1 [file pathogens-14-01189-s001.zip › pathogens-3924184-supplementary.pdf]

SUPPLEMENTAL INFORMATION

Integrating GPC3 With Other Biomarkers to Improve the Diagnosis of Early-Stage Liver Cancer

Authors

Jing Xu [1,2], Lin Tan [2], Ning Jiang [2], Feng Zhang [3], Jinling Wang [2], Fengcheng Li [2], Jin Wang [2], Heng Li [4], Lichang Chen [3], Olivia Mezzetti [5], Wenyu Lin [5,*], Shasha Li [2,*], Yufeng Gao [1,*]

[1] Department of Infectious Diseases, The First Affiliated Hospital of Anhui Medical University, Hefei 230022, Anhui Province, PR China; fyey2563945@163.com (JX)

[2] Department of Hepatology, The Second People's Hospital of Fuyang City, Fuyang 236015, Anhui Province, PR China; tanl558@163.com (LT); jn56857676@163.com (NJ); wjling669@163.com (Jinling W); 879384481@qq.com (FL); wj18940264409@163.com (JW)

[3] Department of Clinical Laboratory, The Second People's Hospital of Fuyang City, Fuyang 236015, Anhui Province, PR China; fuyangeryuanzf@126.com (FZ); 95240337@qq.com (LC)

[4.] Department of Hepatobiliary Surgery, The Second People's Hospital of Fuyang City, Fuyang 236015, Anhui Province, PR China; 249072669@qq.com (HL)

[5.] Liver Center and Gastrointestinal Division, Department of Medicine, Massachusetts General Hospital, Harvard Medical School, Boston, MA 02114, USA; omezzetti@mgh.harvard.edu (OM)

* Correspondence: lishasha20080808@163.com (SL); aygyf@126.com (YG); wlin1@mgh.harvard.edu (WL)

Table S1. Clinical application of different cirrhosis and HCC tumor markers

Marker Name	Clinical Application Points
AFP（Alpha-Fetoprotein）	Sensitivity 40%-65%, specificity 80%-90%, benign liver disease (such as cirrhosis) is prone to false positives.
AFP-L3%（AFP-Lens Culinaris Agglutinin-3 Percentage）	Sensitivity ranges from 42% to 70%, showing insufficient sensitivity for small liver cancers ≤2 cm. With a specificity of 85% to 95% (superior to total AFP), it can effectively differentiate HCC from benign liver diseases.
DCP/PIVKA-II（Des-γ-Carboxyprothrombin/Protein Induced by Vitamin K Absence-II）	Sensitivity: 48%-62%, specificity: 80%-90%; the false-positive rate for benign liver diseases is lower than that of AFP. Significant diagnostic value for AFP-negative HCC.
GPC3（Glypican-3）	With a sensitivity of 50%-72% and specificity of 81%-85%, it can distinguish early-stage HCC from cirrhosis. It shows no correlation with AFP and is suitable for detecting AFP-negative HCC.
G-test（GlycoHCCTest）	This screening is suitable for chronic hepatitis B and cirrhosis patients, supplementing AFP-negative or ≤3cm microcancer detection. It cannot serve as a standalone diagnostic tool and requires combined testing. The technology is complex and requires specialized equipment, with insufficient long-term validation data from large multicenter studies.
Liver Cancer-Related miRNA Panel	Sensitivity: 68.6%, specificity: 90.1%; primarily for high-risk groups with HBV-related liver cancer, heavily dependent on testing technology and platforms, with limited data on early-stage microcancer detection sensitivity.

Table S2. A comparison of the GDATA and GALAD models for HCC diagnosis

Index	GDATA model	GALAD model
Background and purpose	To address the diagnostic needs for early-stage HCC (BCLC 0/A stage) and compensate for the limitations of single biomarkers (e.g., AFP, GPC3).	Developed based on Western populations, this approach aims to enhance the diagnostic efficacy of HCC (including early-stage) through multi-marker integration, addressing the issues of missed or misdiagnosis associated with single-marker testing.
Component indicators	5 items: GPC3, DCP, AFP-L3%, TBIL, age	5 items: age, gender (male = 1, female = 0), AFP, AFP-L3%, DCP
Early HCC diagnostic efficacy	AUC=0.885（95% CI0.827–0.929）<br>accuracy= 81.90%	AUC=0.853（95% CI0.791–0.903）<br>accuracy = 74.72%
Add liver function indicators	Yes (TBIL reflects hepatic metabolic conversion function, compensating for traditional models' neglect of liver function status)	No (relies solely on tumor markers and demographic indicators, without considering the impact of liver function differences on diagnosis)
Research cohort characteristics	Single-center prospective cohort study (Fuyang Second People's Hospital) enrolled 200 subjects (100 with HCC, 100 with CLD), primarily comprising individuals infected with HBV.	Based on Western population cohorts, subsequently validated across multiple regions (including Asian populations), with broader etiological coverage (HBV, HCV, alcoholic liver disease, etc.).

Table S3. Sensitivity of GDATA, GALAD, and single markers at 90% and 95% specificity

**Index**	**Sensitivity and 95% confidence interval at 90% specificity**	**Sensitivity and 95% confidence interval at 95% specificity**
GDATA model	72.46 (52.19 - 85.51)	49.28 (24.64 - 66.67)
GALAD model	60.00 (38.72 - 74.00)	45.00 (28.00 - 67.00)
GPC3	40.58 (28.99 - 55.07)	26.09 (10.14 - 43.48)
DCP	56.52 (45.14 - 73.00)	47.83 (24.64 - 62.32)
AFP	57.97 (39.13 - 76.63)	37.68 (23.19 - 56.52)
AFP-L%	37.68 (26.3%~49.1%)	37.68 (26.09 - 55.07)
